# Supplementary material for: Spatiotemporal control of signal-driven enzymatic reaction in artificial cell-like polymersomes
Source: Nat Commun. 2022 Sep 2;13:5179. doi: 10.1038/s41467-022-32889-7 (PMC9440086; doi:10.1038/s41467-022-32889-7)
Supplement: Supplementary file 1 — Supplementary Information [file 41467_2022_32889_MOESM1_ESM.pdf]

Supplementary Information

Spatiotemporal Control of Signal-Driven Enzymatic Reaction in Artificial Cell-Like Polymersomes

*Hanjin Seo, Hyomin Lee**

H. Seo, Prof. H. Lee
Department of Chemical Engineering
Pohang University of Science and Technology (POSTECH)
77 Cheongam-Ro, Nam-Gu, Pohang, Gyeongbuk 37673, Korea
E-mail: hyomin@postech.ac.kr

Supplementary Discussion

Detailed derivation for determination of ion permeability

To estimate the ion permeability, we adopt the membrane transport equation to describe the ion flux through a thin polymersome membrane as follows.[1-4]

$$\frac{dC_{ions}}{dt} = \frac{P * A}{V} * \Delta C \qquad (1)$$

where C is concentration, P is permeability, A is surface area, and V is volume. By multiplying both sides by V, equation (1) can be re-written in terms of total number of ions.

$$\frac{dN_{ions}}{dt} = P * A * \Delta C \qquad (2)$$

As the influx of water molecules through the membrane is much faster than the permeability of ions, ion permeation can be regarded as the rate-determining step in the overall process. Accordingly, the equation for the water influx to the polymersome can be replaced with the following equation (3) and (4) which describe the instantaneous osmotic equilibrium of the polymersome due to difference in osmolality.[3]

$$C_{ions}^{outer} = \frac{n_{ions}^{vesicles} + n_{PEG}^{vesicles}}{V^{vesicles}} \qquad (3)$$

$$C_{ions}^{outer} * V^{vesicles} = n_{ions}^{vesicles} + n_{PEG}^{vesicles} \qquad (4)$$

Consequently, both sides of equation (4) can be differentiated with respect to time. Since the volume of the outer continuous phase is remarkably large compared to the volume within the polymersomes, we assumed that the initial concentration of the ions would be constant. Accordingly, following equation (5) can be obtained.

$$C_{ions}^{eq} * \frac{dV^{vesicles}}{dt} = \frac{dn_{ions}^{vesicles}}{dt} \quad (\because C_{ions}^{outer} \sim C_{ions}^{eq}, n_{PEG}^{vesicles} = const) \qquad (5)$$

Substituting the right-hand side of equation (5) with equation (2) yields the following expressions.

$$C_{ions}^{eq} * \frac{dV^{vesicles}}{dt} = P * A * \Delta C \qquad (6)$$

$$\frac{dV^{vesicles}}{dt} = \frac{P * A * \Delta C}{C_{ions}^{eq}} = \frac{P * A}{C_{ions}^{eq}} * \frac{n_{PEG}^{vesicles}}{V} \qquad (7)$$

$$4\pi R^2 * \frac{dR}{dt} = P * \frac{4\pi R^2}{C_{ions}^{eq}} * \frac{n_{PEG}^{vesicles}}{\frac{4\pi R^3}{3}} \qquad (8)$$

$$\frac{dR}{dt} = 3 * \frac{P_{ions}}{C_{ions}^{eq}} * \frac{n_{PEG}^{vesicles}}{4\pi R^3} \qquad (9)$$

Integrating both sides with respect to time allows acquisition of the final equation (10) below which relates the ion permeability to normalized polymersome radius change with respect to time.

$$\left(\frac{R}{R_0}\right)^4 = 1 + \frac{4 * P_{ions}}{R_0} * t \qquad (10)$$

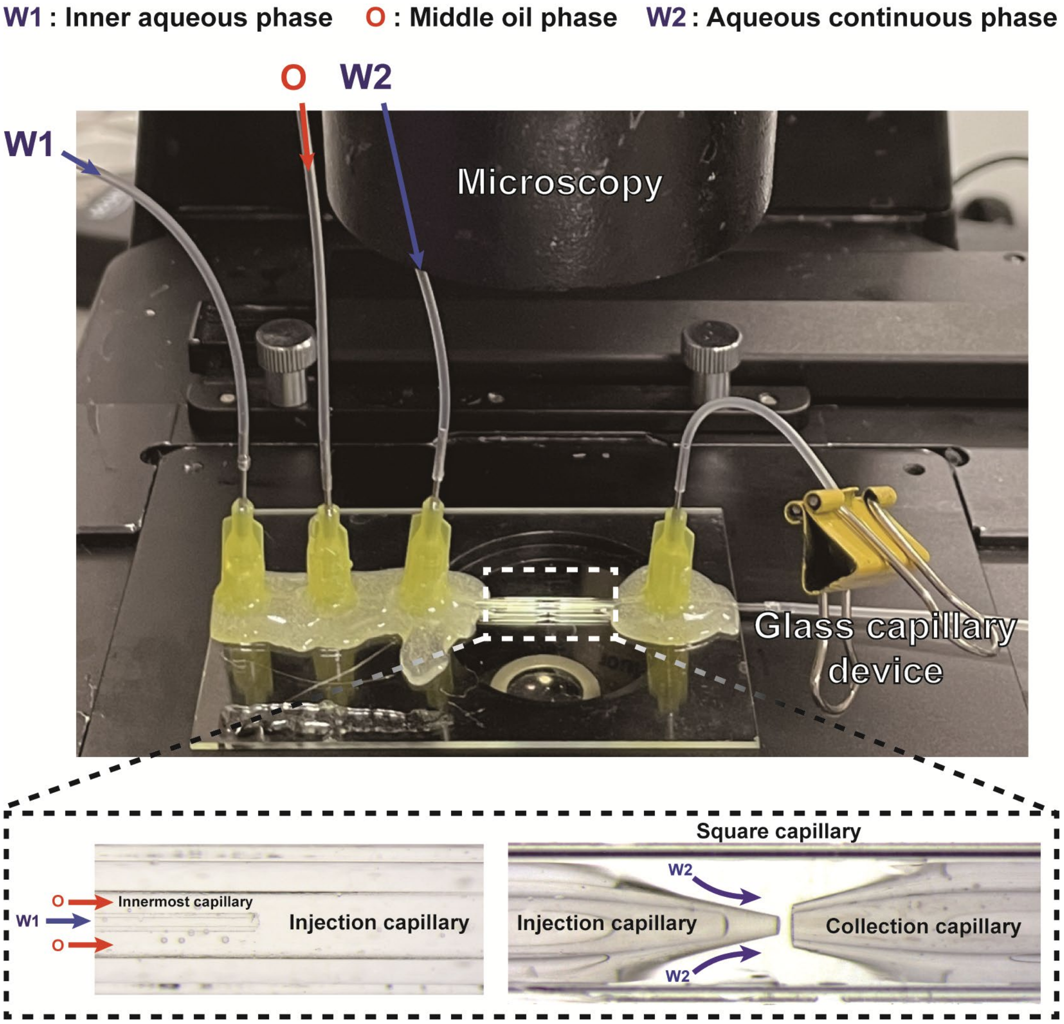

Supplementary Figure 1. Photograph of the device and the detailed setup used for production of Pluronic-based polymersomes.

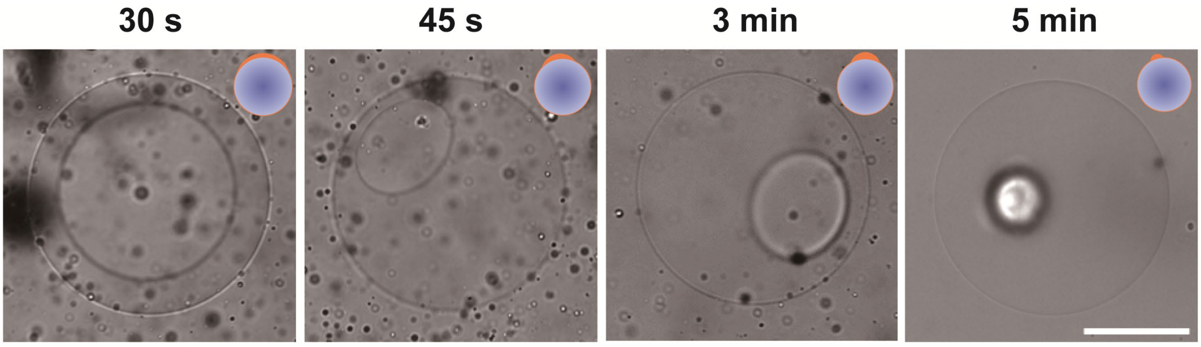

Supplementary Figure 2. Series of optical micrographs showing the dewetting transition of Pluronic-based polymersomes. Scale bar represents 50 μm.

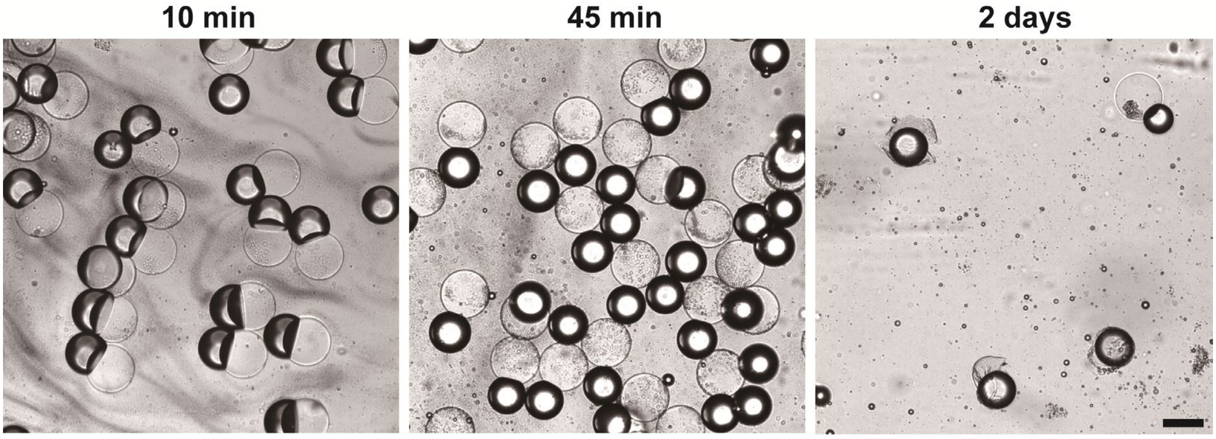

Supplemenatry Figure 3. Series of optical micrographs showing the dewetting transition and rupture over time for lipid-based giant unilamellar vesicles (GUVs). Scale bar represents 100 μm.

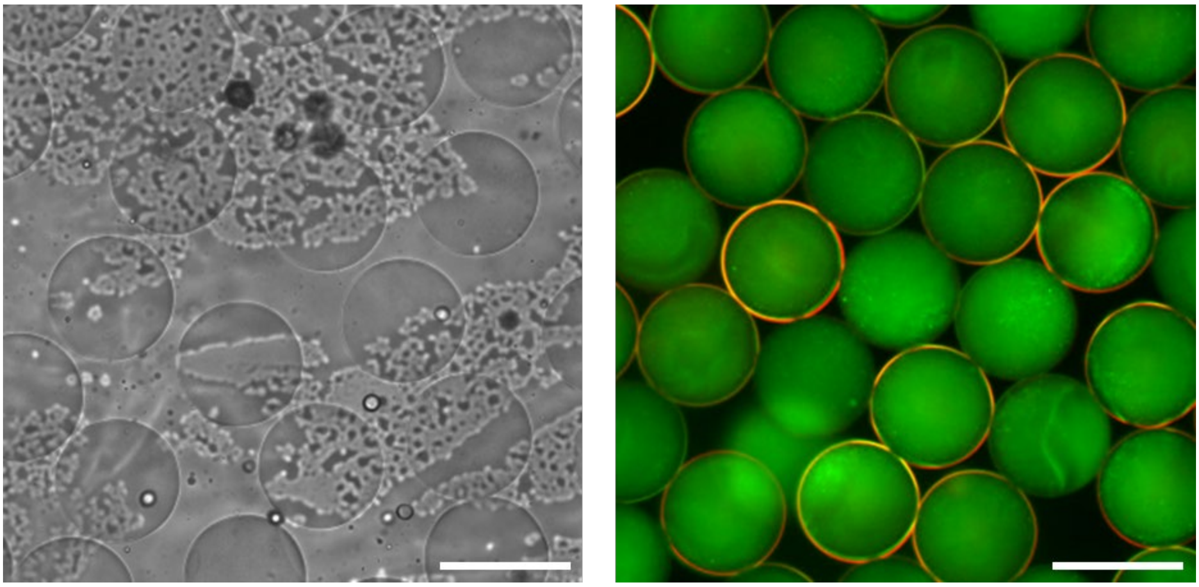

Supplementary Figure 4. Optical and fluorescence micrograph of the Pluronic-based polymersomes after 1 week of incubation in a temperature-adjustable chamber set at 37.0 ± 0.5 °C. Scale bar represents 100 μm.

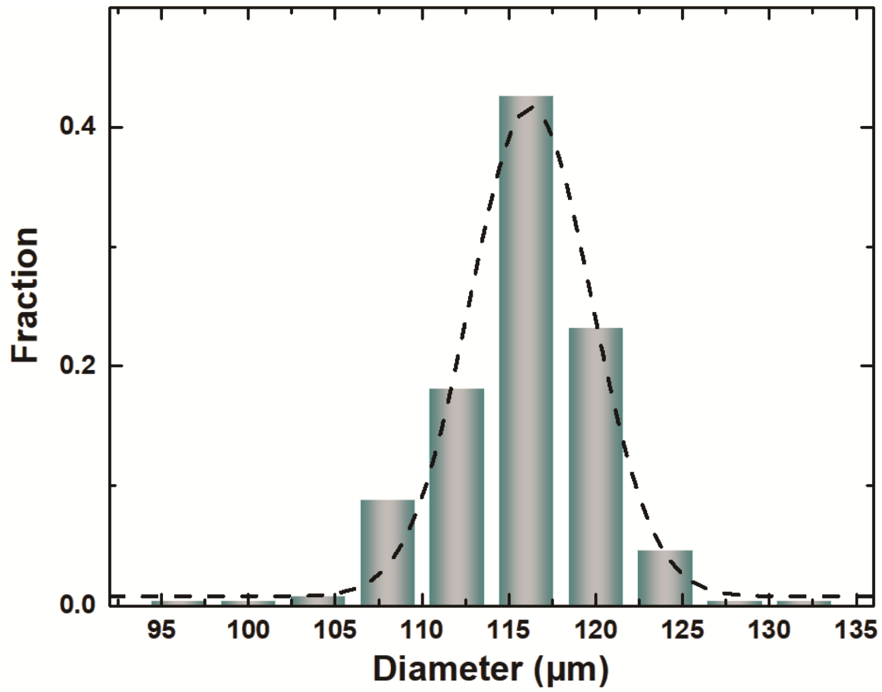

Supplementary Figure 5. Histogram showing the overall diameter distribution of the Pluronic-based polymersomes (n=237).

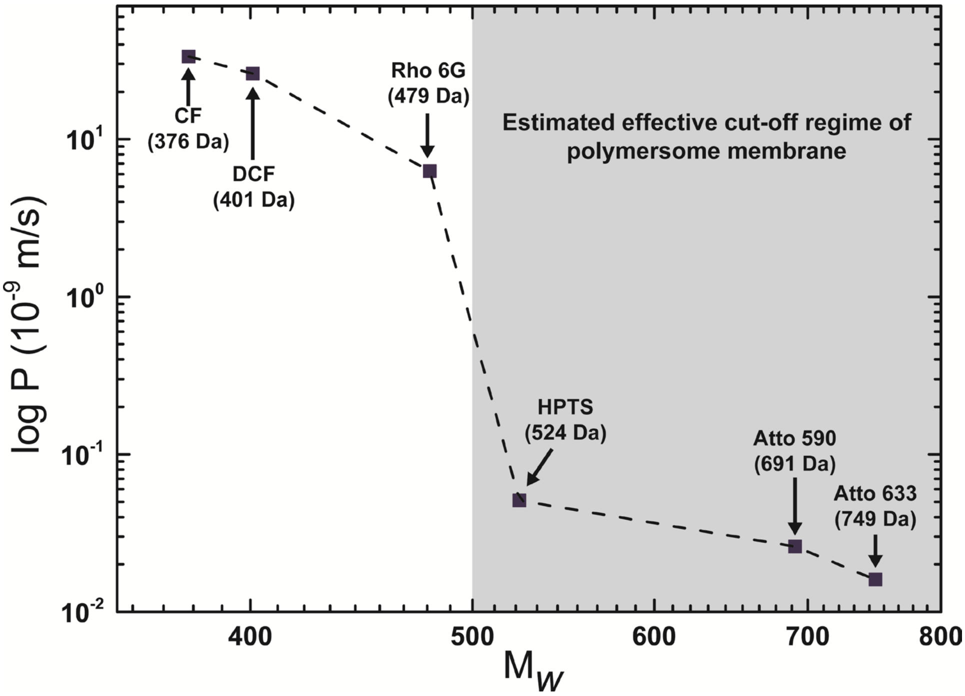

Supplementary Figure 6. A plot showing the estimated permeability of various fluorescent dye molecules with respect to their molecular weight.

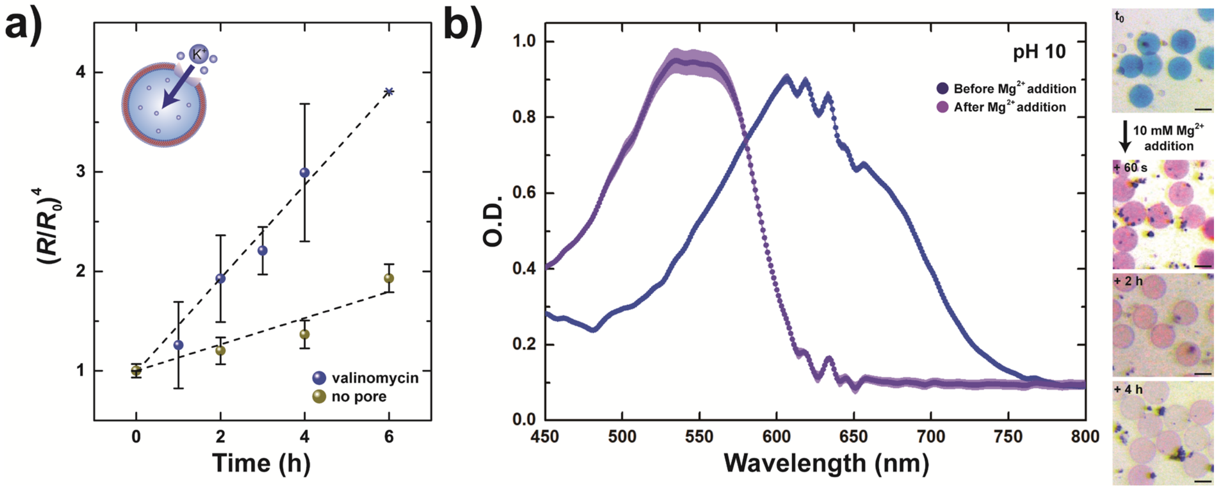

Supplementary Figure 7. a) Plot showing the polymersome volume swelling with respect to time before and after inclusion of K^+ ion-selective channel, valinomycin, in the polymersome membrane (n=11), compared to polymersomes without channel insertion (n=13). All error bars represent standard deviation. b) Series of optical micrographs and an optical density plot each showing the color change and maximal wavelength absorbance peak shift upon injection of 10 mM of Mg^{2+} in the vicinity of polymersomes. Error bands in (b) represent standard deviation, in three independent experiments.

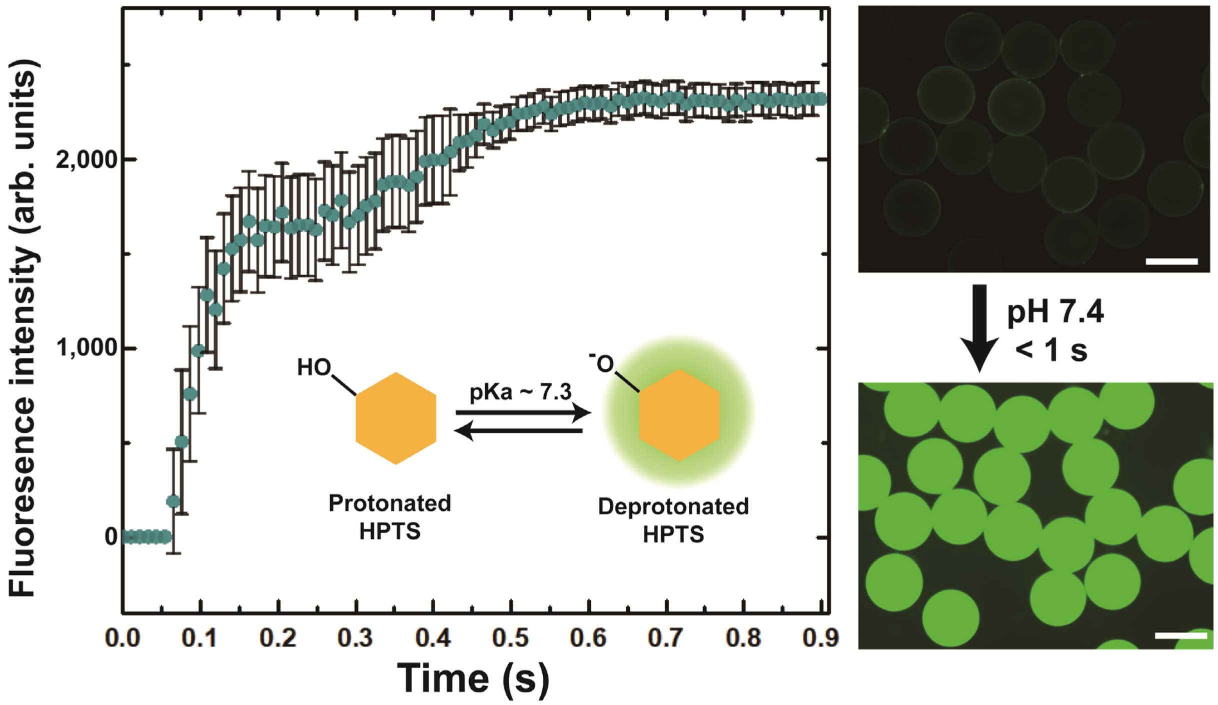

Supplementary Figure 8. Plot and schematic illustration showing the fluorescence intensity evolution over time upon increasing the pH condition to pH 7.4 (n=21). Scale bars in the fluorescence micrographs represent 100 μm. All error bars represent standard deviation.

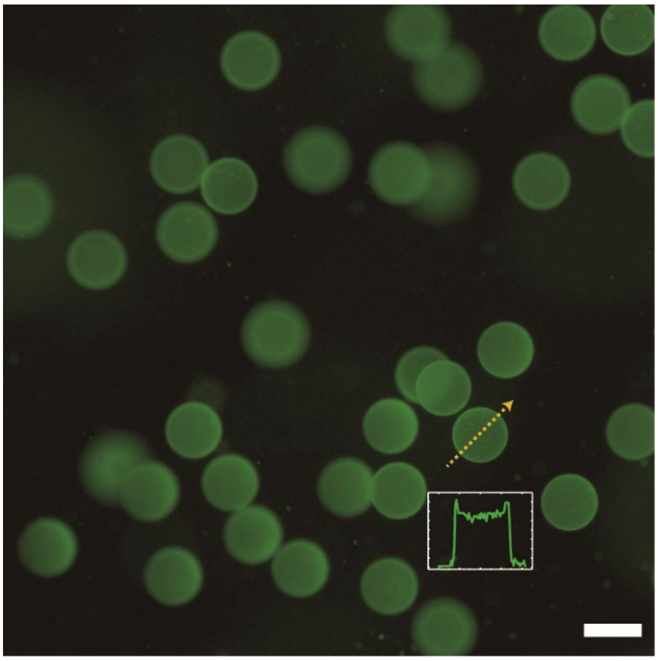

Supplementary Figure 9. Fluorescence micrograph of the polymersomes containing an aqueous solution of 10 mg mL^{-1} PAH and 0.5 mg mL^{-1} FITC-PAH (pH 4.0), which are subjected for 24 h in a pH 8 adjusted aqueous media containing 10 mM ATP solution. Inset plot shows the spatially resolved intensity profiles of the green fluorescence in the polymersomes. Scale bar represents 100 µm.

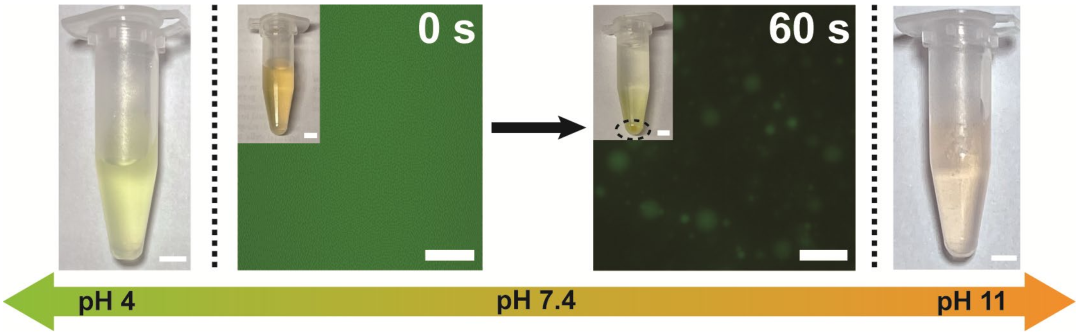

Supplementary Figure 10. Photographs and fluorescence micrographs showing the pH-responsive complex coacervate formation. Scale bars represent 5 mm for the centrifuge tubes while the scale bars in the fluorescence micrographs are 20 µm. The discrete phase appearing at the bottom of the centrifuge tube after pH adjustment, followed by centrifugation at 1,350 rpm for 10 min reveals that the pH of the media can be altered to modulate the electrostatic interaction between ATP and PAH and induce complex coacervation.

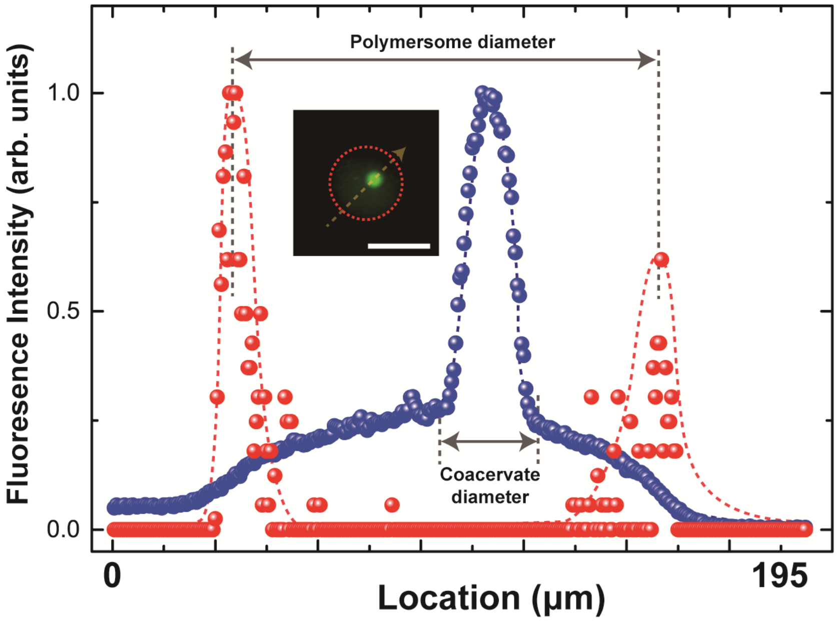

Supplementary Figure 11. A plot showing the spatially resolved intensity profiles of the matured single large coacervate droplet within the polymersome at 10 mM ATP after 120 min. Scale bar in the inset represents 100 µm.

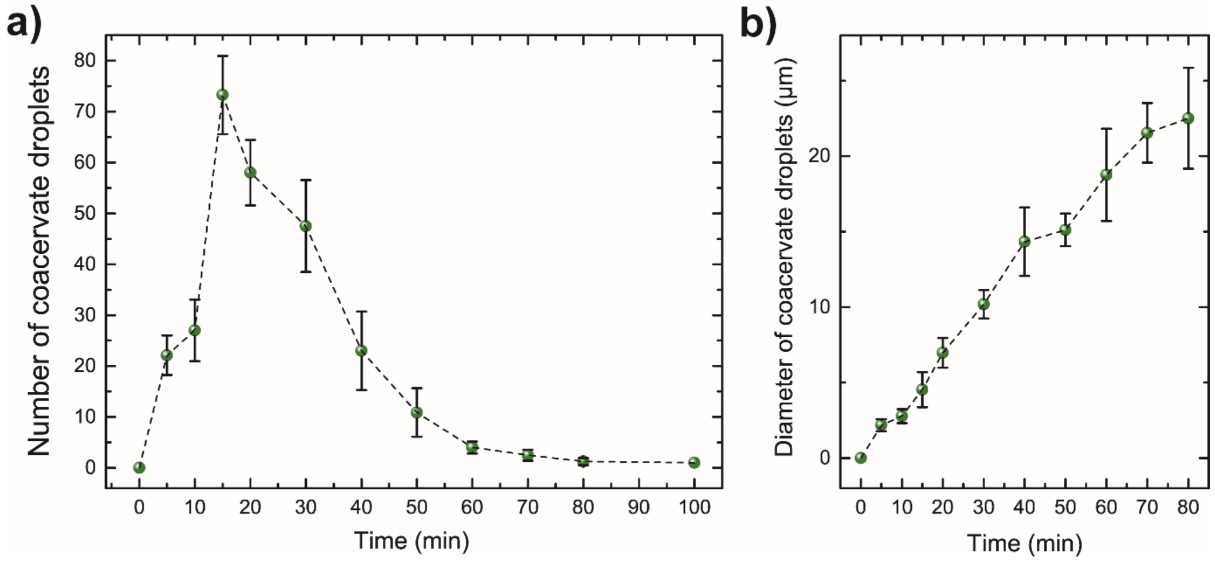

Supplementary Figure 12. a) Plot showing the change in the number of coacervate droplets inside polymersomes after increasing the pH condition to pH 9.45. (n=7). b) Plot showing the average diameter of the matured coacervate (t=120 min) inside the polymersomes with time (n=5). All error bars represent standard deviation.

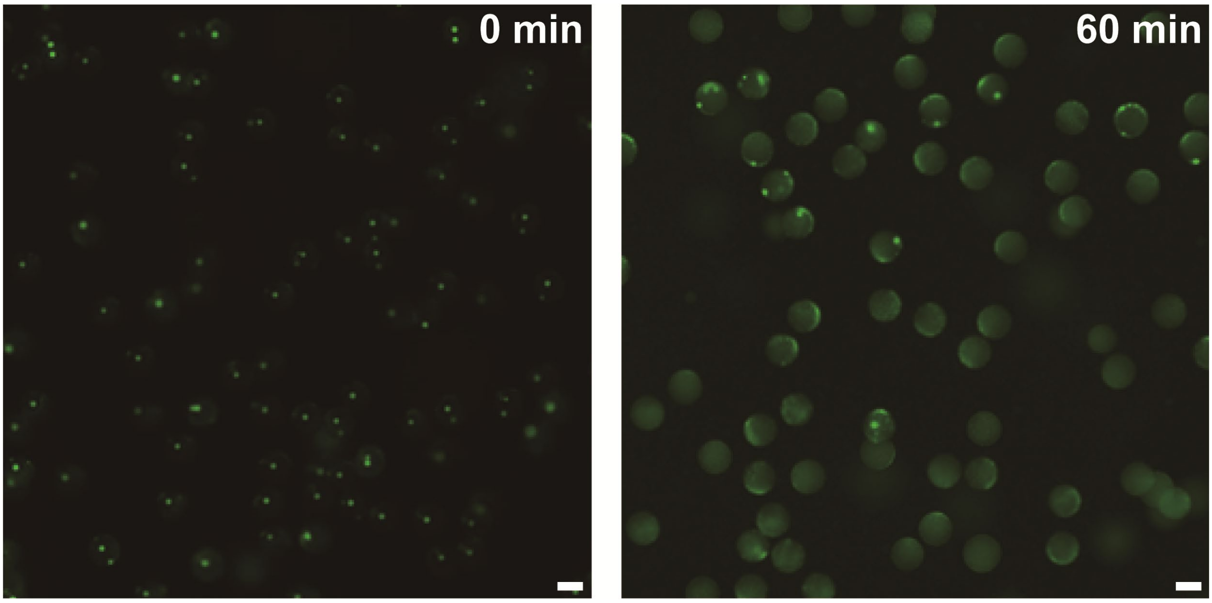

Supplementary Figure 13. Fluorescence micrographs of polymersomes (10 mM ATP and 10 mg mL^{-1} PAH) before and after increasing the pH condition from 7.4 to pH 11. Scale bar represents 100 μm.

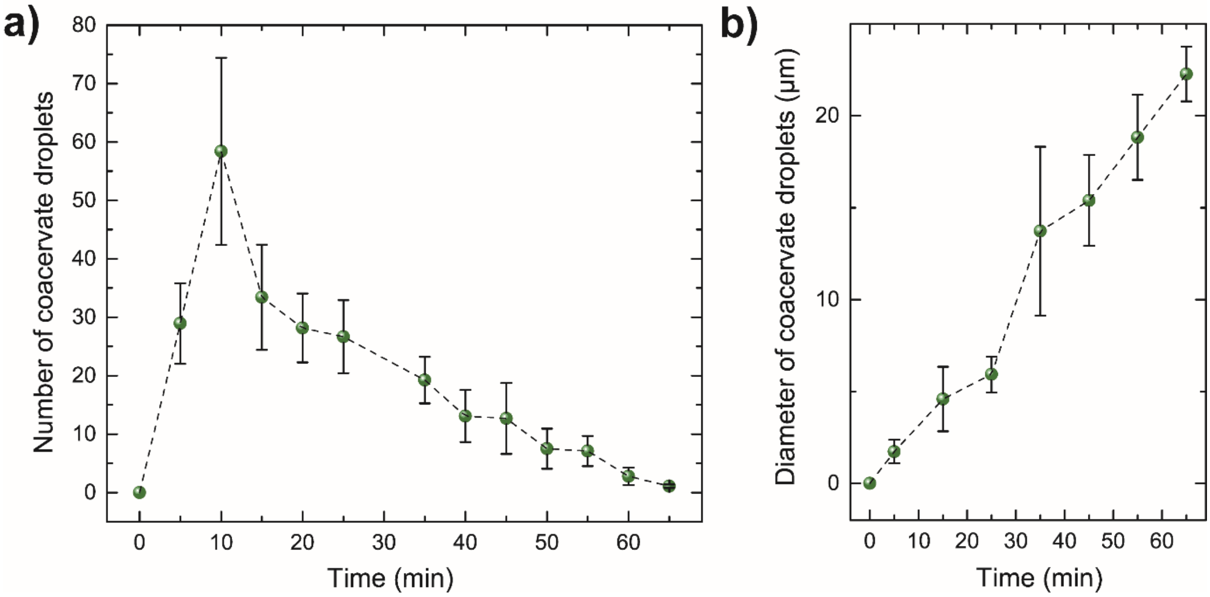

Supplementary Figure 14. a) Plot showing the change in the number of coacervate droplets inside smaller-sized polymersomes (10 mM ATP, 10 mg mL^{-1} PAH) with diameter of 78.91 ± 4.54 μm after increasing the pH condition to pH 7.4. (n=12). b) Plot showing the change in the diameter of the matured coacervate inside these polymersomes with time (n=5). All error bars represent standard deviation.

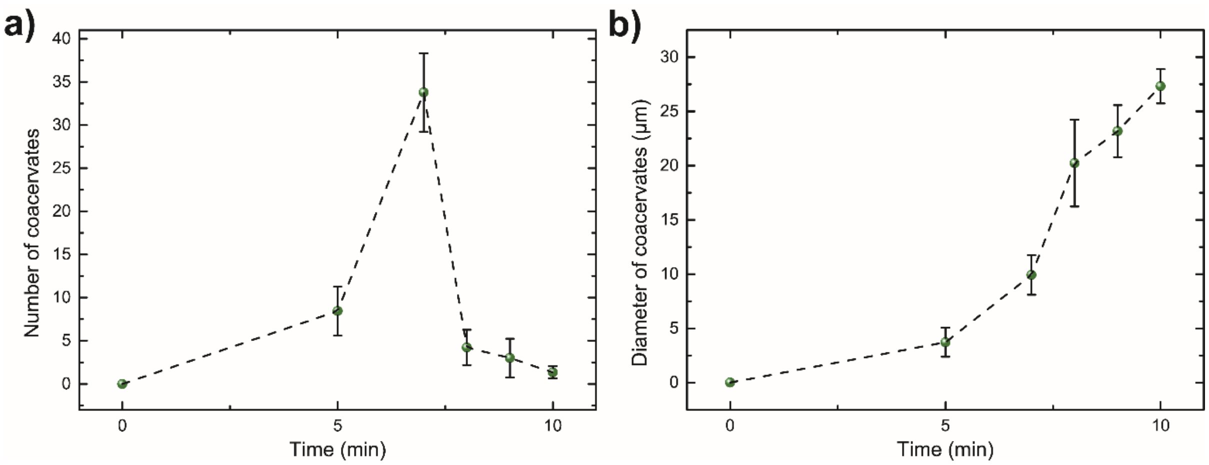

Supplementary Figure 15. a) Plot showing the change in the number of coacervate droplets inside polymersomes (10 mM ATP, 10 mg mL^{-1} PAH) after lowering the pH condition from 10.5 to pH 7.4. (n=9). b) Plot showing the change in the diameter of the matured coacervate inside these polymersomes with time (n=9). All error bars represent standard deviation.

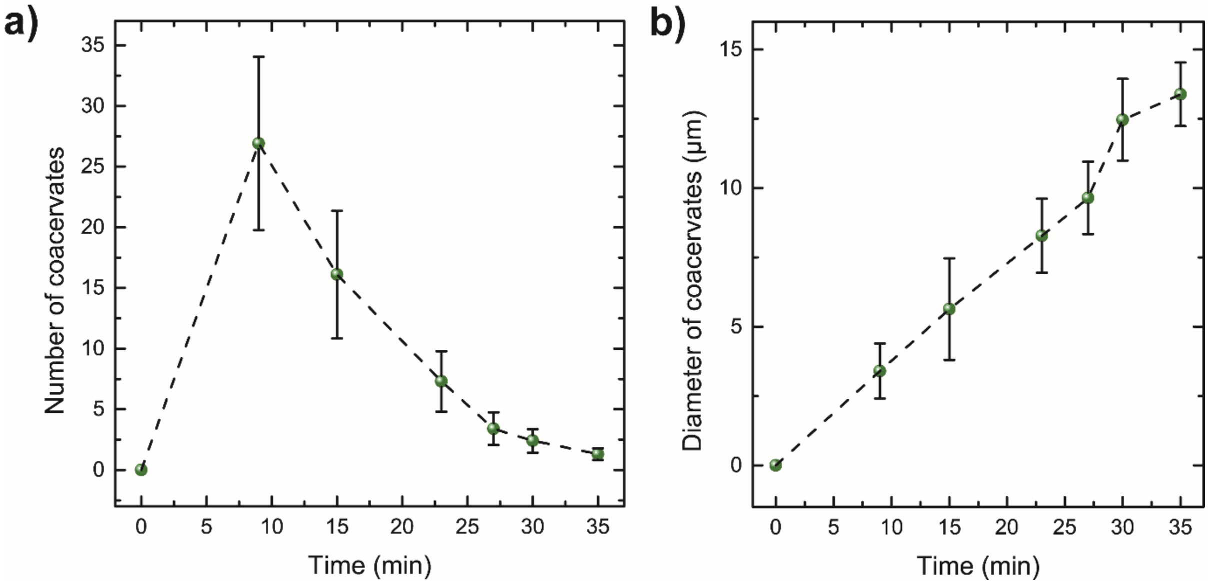

Supplementary Figure 16. a) Plot showing the change in the number of coacervate droplets inside polymersomes (10 mM ATP and 5 mg mL^{-1} PAH) after increasing the pH condition from 4 to pH 7.4. (n=10). b) Plot showing the change in the diameter of the matured coacervate inside these polymersomes with time (n=10). All error bars represent standard deviation.

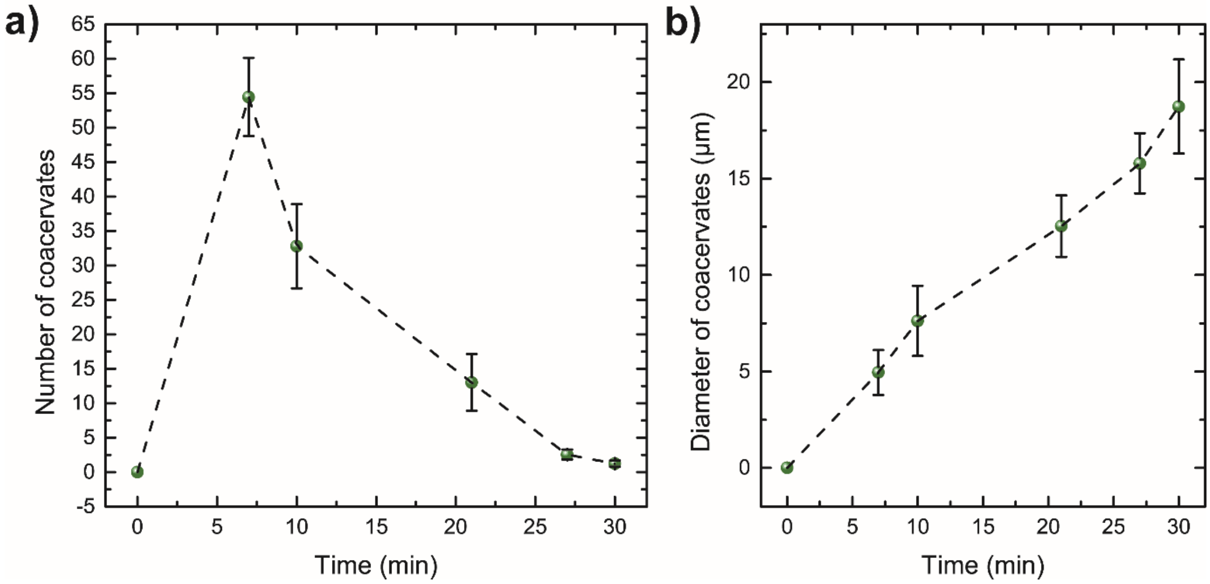

Supplementary Figure 17. a) Plot showing the change in the number of coacervate droplets inside polymersomes (5 mM ATP, 10 mg mL^{-1} PAH) after lowering the pH condition from 10.5 to pH 7.4. (n=9). b) Plot showing the change in the diameter of the matured coacervate inside these polymersomes with time (n=9). All error bars represent standard deviation.

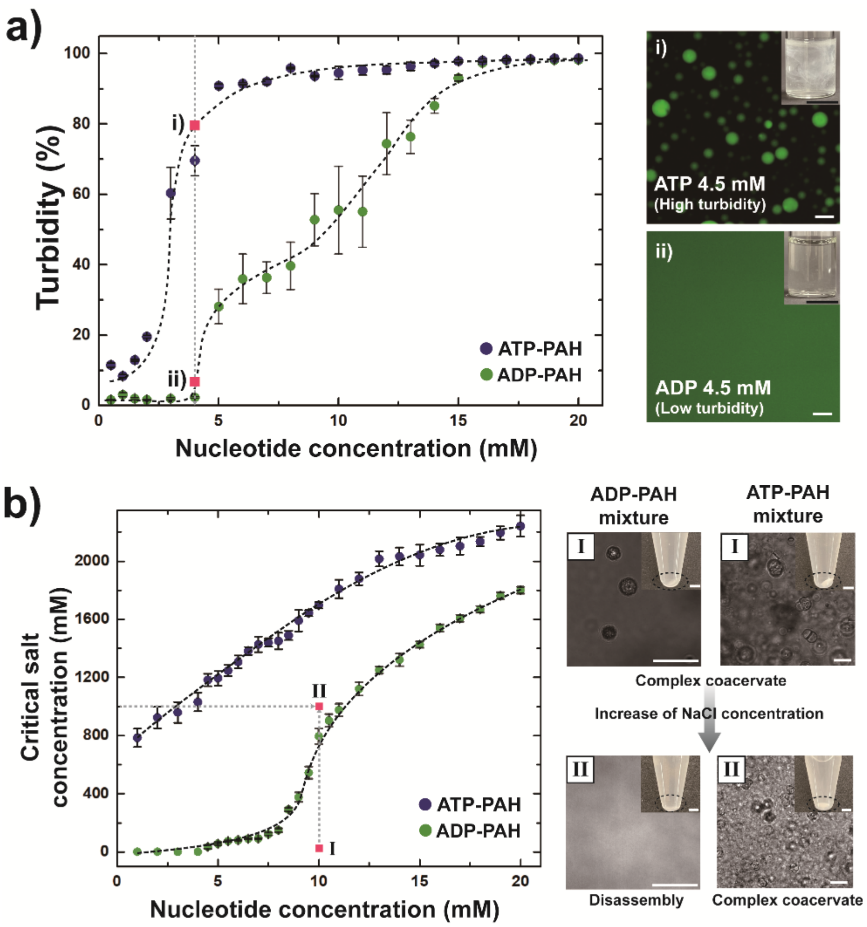

Supplementary Figure 18. a) A plot showing the turbidity value of the mixture containing nucleotides and PAH. PAH concentration is fixed at 10 mg mL^{-1} while the concentration of nucleotides is varied from 0 to 20 mM. Green data points (with dashed line as a guide to the eye) in the plot represent the ADP-PAH mixture while the blue data points (with dashed line as a guide to the eye) represent ATP-PAH mixture. Photographs and the fluorescence micrographs of these mixtures at the same nucleotide concentration of 4.5 mM with additional 0.5 mg mL^{-1} of green fluorescent dye labeled PAH (FITC-PAH) is shown on the right. i) ATP-PAH mixture, ii) ADP-PAH mixture. Black scale bars in the photographs represent 10 mm while the white scale bars in the fluorescence micrographs are 20 µm; n=10 at each concentration point. Data are presented as mean values +/- standard deviation. b) Critical salt concentration plot for complex coacervate comprising of either ADP-PAH (green data points) or ATP-PAH (blue data points) each with dashed line as a guide to the eye. The point I refers to 10 mM nucleotide concentration with no additional NaCl, while point II refers to the same nucleotide concentration but with additional 1M NaCl. (I) With no additional NaCl, both ADP and ATP form coacervates with PAH. (II) With addition of 1M NaCl, ADP-PAH coacervate disassembles while ATP-PAH coacervate maintains. Photographs and the optical micrographs of the ADP-PAH and ATP-PAH mixture at these two points. Scale bars in the optical micrographs are 10 µm while the scale bars represent 3.5 mm for photographs; n=10 at each concentration point. Data are presented as mean values +/- standard deviation.

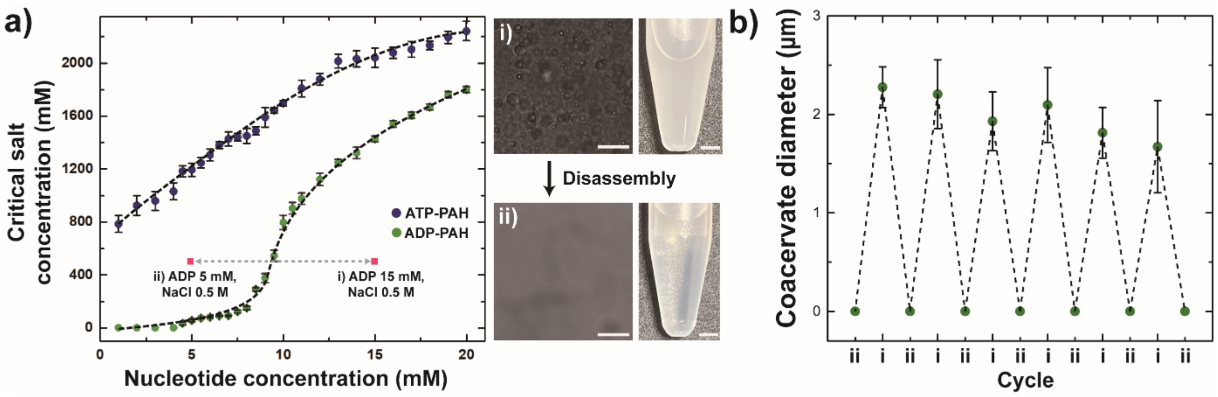

Supplementary Figure 19. a) Critical salt concentration plot showing the transition from ADP concentration of i) 15 mᴍ and ii) 5 mᴍ at a fixed NaCl concentration of 0.5M. Optical micrographs and photographs showing the disassembly of ADP-PAH coacervate upon reducing the ADP concentration to 5 mᴍ. Scale bar in the optical micrograph represents 10 µm while the scale bar in the photograph represents 5 mm; n=10 at each concentration point. Data are presented as mean values +/- standard deviation. b) Reversibility plot showing the average ADP-PAH coacervate droplet size as the ADP concentration is modulated from i) 15 mᴍ to ii) 5 mᴍ. Data are presented as mean values +/- standard deviation, and n=36 at each cycle point.

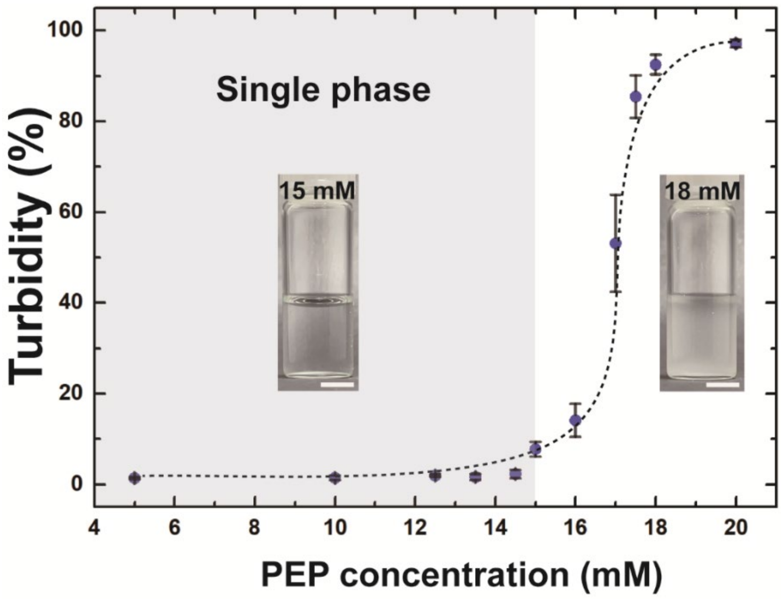

Supplementary Figure 20. A plot showing the turbidity value of the mixture containing PEP and PAH. PAH concentration is fixed at 10 mg mL^{-1} while the concentration of PEP is varied from 5 to 20 mᴍ. The mixture appears as a single phase at concentrations below 15 mᴍ (grey region) and high turbidity is observed above 18 mᴍ. The inset photographs show the PEP-PAH mixture at PEP concentrations of 15mᴍ and 18 mᴍ, respectively. Scale bars in the inset represent 7 mm; n=7 at each concentration point. All error bars represent standard deviation.

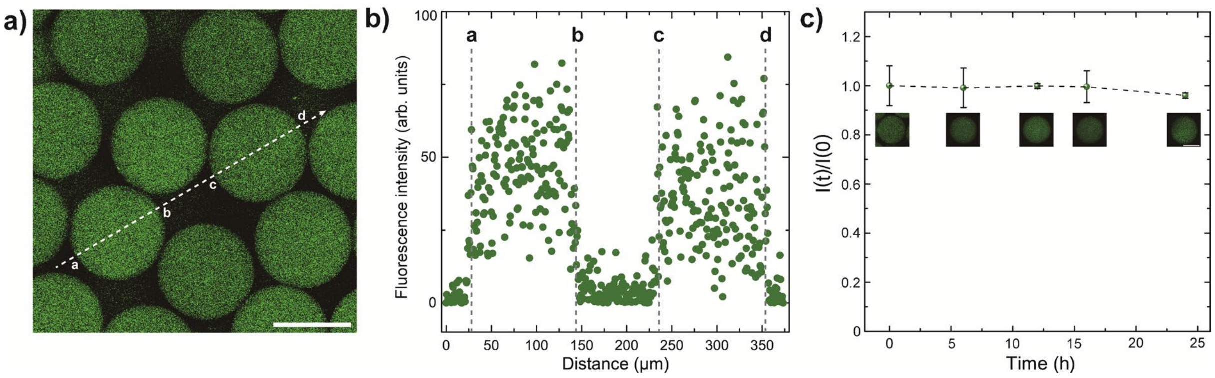

Supplementary Figure 21. (a) Fluorescence micrograph showing the Pluronic-based polymersomes encapsulating Fluorescein labeled pyruvate kinase (FITC-PyK). Scale bar represents 100 µm. (b) Plot represents the spatially resolved intensity profile across the polymersome interior and exterior. (c) Plot and fluorescence micrographs showing the steady fluorescence intensity within the polymersome. (n=5) Scale bar represents 100 µm. All error bars represent standard deviation.

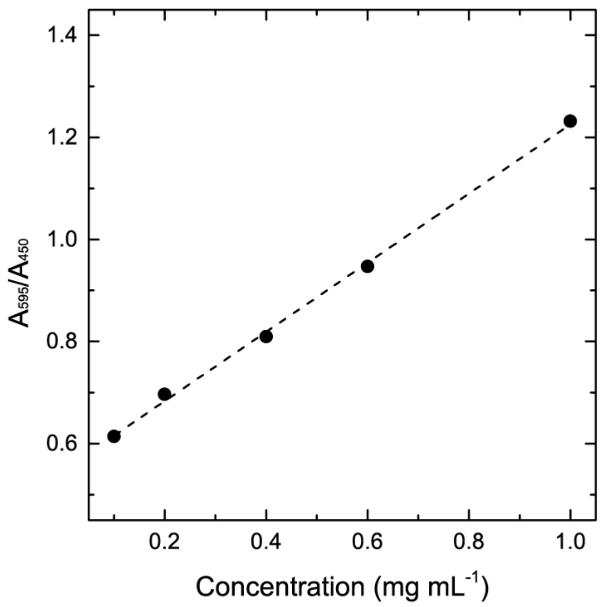

Supplementary Figure 22. Linearized standard curve of BSA solution (0.1 – 1.0 mg mL^{-1}). The fitted equation yields, Y = 0.67776*X + 0.54779 with R^2=0.99798.

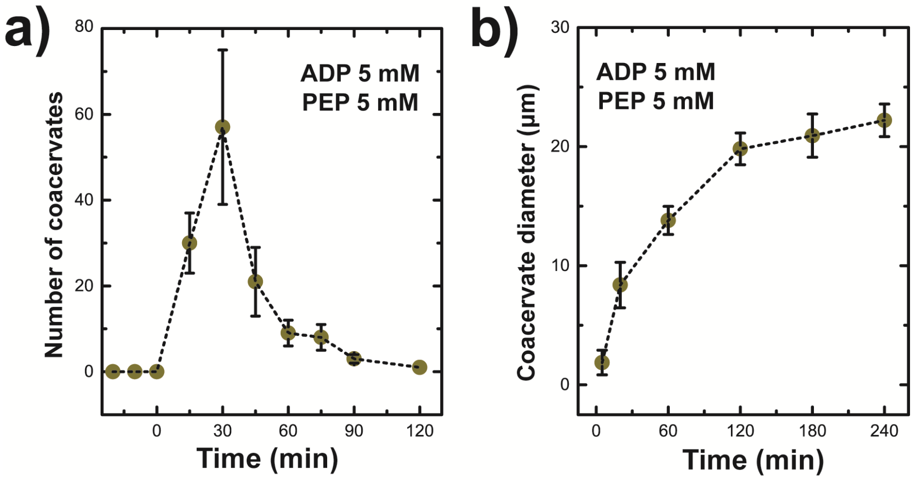

Supplementary Figure 23. (a) Plot showing the change in the number of coacervate droplets inside polymersomes (n=9). (b) Plot showing the evolution of average coacervate droplet diameter inside polymersomes. (n=10) Data are presented as mean values +/- standard deviation.

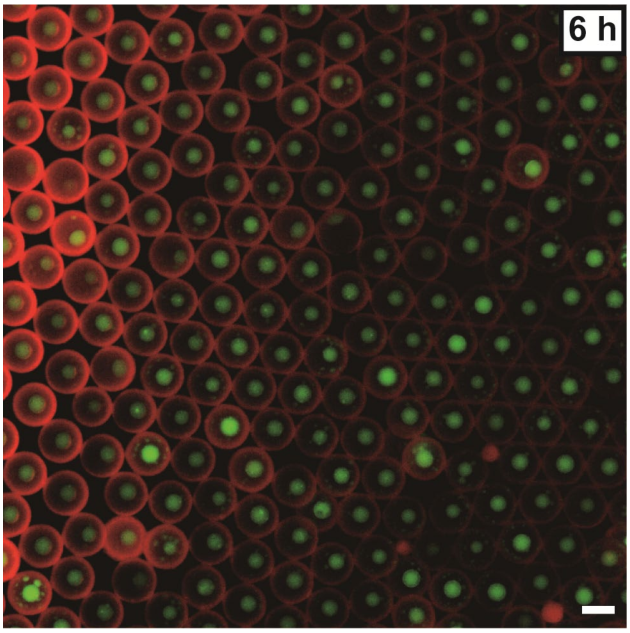

Supplementary Figure 24. Fluorescence micrograph showing the complex coacervate formation within polymersomes 6 h after infusing PEP. Scale bar represents 100 µm.

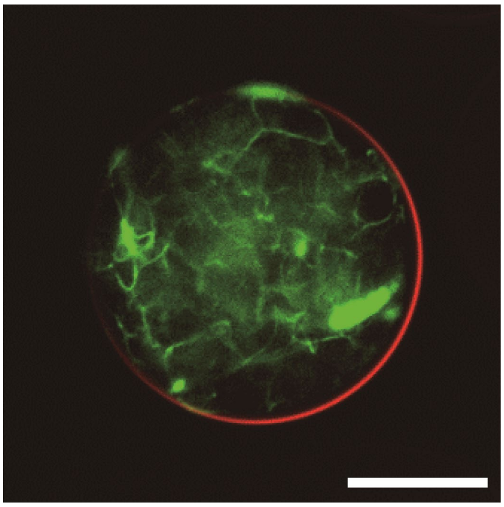

Supplementary Figure 25. Confocal micrograph showing the F-actin formation in a Pluronic-based polymersome. Scale bar represents 50 μm.

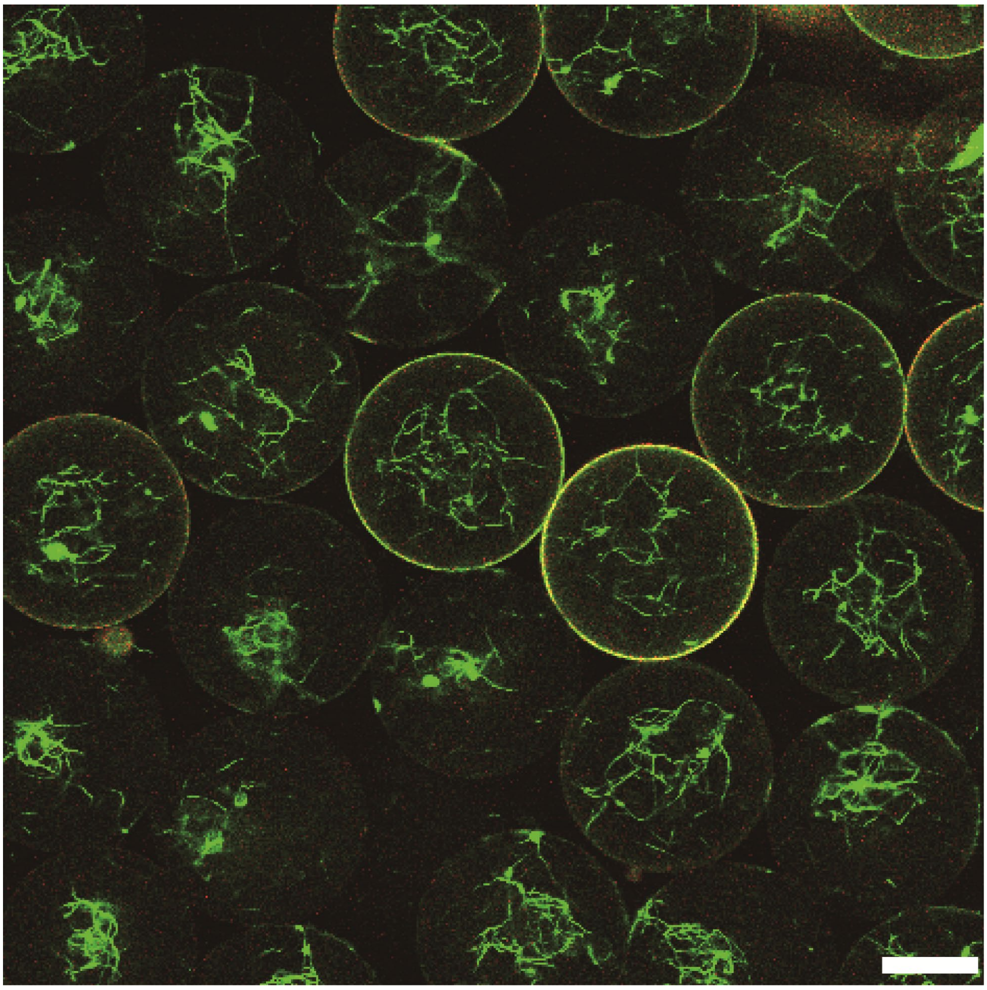

Supplementary Figure 26. Low magnification confocal micrograph showing the actin filament formation by signal-driven actin polymerization in Pluronic-based polymersomes. Scale bar represents 50 μm.

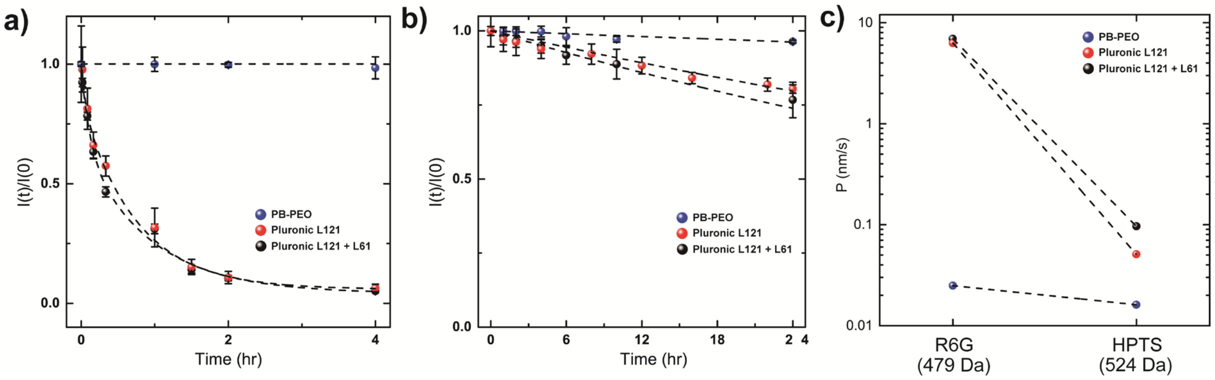

Supplementary Figure 27. (a) Plot showing the normalized fluorescence intensity change over time within the three sets of polymersomes with different compositions using Rhodamine 6G (R6G). Blue dot refers to PB-PEO polymersome (n=10), while the red and black dot refers to Pluronic L121-based polymersome (control, n=11) and Pluronic L121 + L61 (75:25 mol%) blended polymersome (n=11). (b) Plot showing the normalized fluorescence intensity change over time within the sets of polymersomes with different compositions using HPTS. The dotted lines in both plots represent the model fit used to determine the membrane permeability for each diffusing species. (n=10 for PB-PEO polymersomes, n=11 for Pluronic L121-based polymersomes, and n=9 for Pluronic L121+L61 polymersomes) (c) Plot showing the estimated permeability of two fluorescent dye molecules with respect to different compositions in polymersomes. All error bars represent standard deviation, in (a) and (b).

Supplementary Notes

Note for Supplementary Figure 1. Microfluidic device comprises of a square capillary and two tapered cylindrical capillaries, one for the injection, and the other for collection. Prior to inserting and aligning these cylindrical capillaries in a square capillary that has an inner dimension slightly larger than the outer diameter of the cylindrical ones, the inner surface of the injection capillary was modified hydrophobic while the outer surface of the injection capillary as well as the entire collection capillary was made hydrophilic.[5] This allows facile infusion of fluids comprising the double emulsion droplet as well as robust break-up of the biphasic flow at the flow focusing junction. Additionally, another cylindrical capillary whose outer diameter is smaller than the inner diameter of the injection capillary is inserted into the injection capillary to simultaneously inject the inner aqueous phase and the middle oil phase.

Note for Supplementary Figure 3. To fabricate these lipid-based giant unilamellar vesicles, we use a separate glass capillary-based microfluidic device which comprises of a square capillary and two tapered cylindrical capillaries, to form W/O/W double-emulsion template. Cylindrical capillaries with an outer diameter of 1.00 mm (Atlantic International Technology, Inc.) were used for preparing the injection capillary as well as the collection capillary. The inner surface of the injection capillary was treated with 2-[methoxy(polyethyleneoxy)propyl]-trimethoxy silane (Gelest, Inc.) to render the inner surface hydrophilic. The outer surface of the injection capillary was treated with n-octadecyltrimethoxysilane (Sigma Aldrich) to make the outer surface hydrophobic. The both inner and outer surface of the collection capillary were treated with 2-[methoxy(polyethyleneoxy)propyl]-trimethoxy silane to render the both surfaces hydrophilic. Both cylindrical capillaries are tapered using micropipette puller (P-97, Sutter Instrument) and further polished with a sandpaper. Further, these cylindrical capillaries are coaxially aligned in a square capillary with an inner width of 1.05 mm, and fixed to the slide glass with 5 min epoxy (Devcon), prior to inserting and aligning these cylindrical capillaries in a square capillary that has an inner dimension slightly larger than the outer diameter of the cylindrical ones.

Note for Supplementary Figure 7. Acquisition of the polymersome swelling upon inserting 0.1 mg mL^{-1} of K^+ ion-selective channel, valinomycin, into the polymersomes prior to incubation in the hypertonic KCl solution confirms that the polymersomes are indeed

permeable to K^+ and that the permeability can be enhanced by approximately 2.67-folds (P_{K^+} = 1.74 nm s^{-1}) *via* inclusion of ion-channels as shown in Supplementary Fig. 7a. As for the Mg^{2+}, we employ the complexometric titration method in which we incorporate eriochrome black T that changes color upon reaction with divalent metal ions in the aqueous core and inject 10 mᴍ of Mg^{2+} in the vicinity of these polymersomes to separately confirm the permeation of Mg^{2+} through the Pluronic-based membrane (Supplementary Fig. 7b) The change of polymersome color from clear blue to wine color within a minute after injection as well as the shift of maximal optical density peak from 600 to 550 nm clearly shows the complex formation due to inward diffusion of Mg^{2+} through the membrane.

Note for Supplementary Figure 8. To check proton permeability, we prepare a separate set of polymersomes encapsulating 5 µg mL^{-1} HPTS solution adjusted to acidic pH of 4.0. After polymersomes settle in the collection bath, we gently add an aliquot amount of pH 10.0 buffered solution to the periphery of polymersomes to increase the overall pH condition to 7.4.

Note for Supplementary Figure 9. To verify whether ATP (MW 507 Da) can be retained within the membrane, we introduce ATP outside the polymersomes containing only PAH in the aqueous core. We find that even when the polymersomes containing an aqueous solution of 10 mg mL^{-1} PAH and 0.5 mg mL^{-1} FITC-PAH (pH 4) are subjected to pH 8 adjusted media containing 10 mᴍ ATP solution for 24 h, no noticeable formation of complex coacervate is observed, as evidenced by the homogeneous green fluorescence signal within the polymersomes (Supplementary Fig. 9). Consistent with the membrane MW cut-off acquired previously, this indicates that ATP cannot effectively diffuse through the polymersomes' membrane.

Note for Supplementary Figure 10. To verify the pH-controllable complex coacervation between ATP and PAH, we prepare an aqueous solution containing 10 mᴍ ATP and 10 mg mL^{-1} of PAH (MW ~17 500 Da). Additionally, 0.5 mg mL^{-1} of green fluorescent dye labeled PAH (poly(fluorescein isothiocyanate allylamine hydrochloride, FITC-PAH) was added for visualization purposes. We observe no apparent coacervate formation at pH 4 and pH 11, which is either lower than the pKa of ATP or higher than the pKa of PAH. However, at the

intermediate pH of 7.4 at which both ATP and PAH are oppositely charged, complex coacervate forms within a minute.

Note for Supplementary Figure 15-17. We conducted a separate set of experiment in which two sets polymersomes each containing i) 10 mM ATP, 5 mg mL^{-1} PAH, and ii) 5 mM ATP, 10 mg mL^{-1} PAH, respectively, in the aqueous core were prepared to further verify whether the surface charge of the complex coacervate as well as the direction of pH transition indeed affects the time scale of coacervation. As the zeta-potential value of the matured coacervate droplet formed in bulk were +36.8 ± 4.9 mV and +68.0 ± 2.7 mV for i) and ii) respectively, inducing coacervation by increasing the pH from 4 to pH 7.4 for i) will show whether less positive surface charge than the control (10 mM ATP, 10 mg mL^{-1} PAH) will decrease the time scale of coacervation. Conversely, lowering the pH from 10.5 to pH 7.4 for ii) will reveal whether the time scale will be increased compared to the control by increasing the surface charge. In the first case where the pH was raised from 4 to 7.4 for the polymersomes containing 10 mM ATP and 5 mg mL^{-1} PAH, we observe that the overall coacervation process took about 35 min and the average size of the resulting matured coacervate droplet is 13.38 ± 1.15 µm (Supplementary Fig. 16). This clearly shows that these polymersomes exhibit faster coacervation compared to that of polymersomes containing 10 mM ATP and 10 mg mL^{-1} PAH which took more than 1 h in the same direction of pH transition. In the second case in which the pH was lowered from 10.5 to 7.4 for the polymersomes cotainining 5 mM ATP and 10 mg mL^{-1} PAH, we observe that the PAH and ATP matured into a large coacervate droplet after about 30 min and the average size is 18.73 ± 2.44 µm (Supplementary Fig. 17). This confirms that the coacervation time scale has been increased as predicted to exhibit slower coacervation compared to that of polymersomes containing 10 mM ATP and 10 mg mL^{-1} PAH which took about 10 min to complete in the same direction of pH transition.

Note for Supplementary Figure 18, and 19. We investigate the extent of coacervation at pH 7.4 buffer solution in bulk for both nucleotides, ADP and ATP, with respect to PAH using a turbidity assay. We acquire the turbidity value while we fix the PAH concentration at 10 mg mL^{-1} and vary the concentrations of nucleotides from 0 to 20 mM (Supplementary Fig. 18a**)**. Here, relatively high concentration of PAH (10 mg mL^{-1}) was used for the analysis of coacervate formation,[8, 43] as their behavior at lower concentration regime is sensitive and difficult to quantify using the turbidity assay, as reported previously by others.[6, 7]

In the case of ATP, we observe that the turbidity level increases steeply at the concentration of 1.5 mM and reach high turbidity (> 60 %) above 3 mM. On the other hand, no significant change in transmittance is observed for ADP up to 4.5 mM and the turbidity increases gradually with increase in concentration, exceeding the value of 60% above 12 mM, which is 4-folds higher in concentration compared to ATP. Direct comparison between the ATP-PAH and ADP-PAH mixture at the same nucleotide concentration of 4.5 mM with additional 0.5 mg mL^{-1} of green fluorescent dye labeled PAH (FITC-PAH) in the solution clearly reveals that ATP-PAH solution forms complex coacervates and thus is turbid while ADP-PAH solution does not and remain transparent (Supplementary Fig. 18a). These results show that ATP with one more phosphate group than ADP can more effectively interact with PAH *via* electrostatic interactions to form complex coacervates.

To further verify the stronger electrostatic interaction of ATP-PAH compared to ADP-PAH, we investigated the effect of additionally added salt, NaCl, on screening the net electrostatic attraction between both nucleotides and PAH, which leads to disassembly of the preformed coacervates. The buffer solution prior to addition of NaCl includes 150 mM KCl, 25 mM HEPES, and 5 mM $MgCl_2$ to rule out any pH effect and contains metal ion cofactors (K^+, Mg^{2+}) for the enzymatic reaction. We note that the no coacervate formation observed in Supplementary Fig. 18a, ii for ADP-PAH with 4.5 mM ADP was possibly due to these salts that were already included in the buffered solution.

To obtain the critical salt concentration above which the preformed coacervate comprising of either ATP-PAH or ADP-PAH disassembles, we monitor the zero turbidity points for sets of nucleotide/PAH systems with different nucleotide concentrations as concentrated NaCl buffered solution is introduced into each sample. The resulting critical salt concentration plot (Supplementary Fig. 18b) shows that in the entire nucleotide concentration range investigated, the critical salt concentration for ATP-PAH is higher than ADP-PAH. As a result, at the same nucleotide concentration of 10 mM, both nucleotide/PAH system form coacervates without any additional NaCl (Supplementary Fig. 18b, I). However, increasing the NaCl concentration to 1M leads to disassembly of coacervate for ADP-PAH while the coacervate is maintained for ATP-PAH (Supplementary Fig. 18b, II). In addition, when the additional NaCl concentration is fixed at 0.5M, we observe reversible assembly and disassembly of ADP-PAH coacervate with no significant change in the coacervate size as the ADP concentration was modulated from 15 mM to 5 mM (Supplementary Fig. 19). These results indicate that the electrostatic attraction between either ADP or ATP with PAH can be controlled by the amount of added NaCl, as they reduce the net interaction between nucleotide and PAH. Based on this study, we chose 5 mM of

ADP and 0.2 M NaCl as the optimal condition at which ADP does not form coacervate with PAH while equivalent amount of ATP readily forms coacervate with PAH for the demonstration of PEP-driven enzymatic conversion of ADP to ATP, resulting in complex coacervation in polymersomes.

Notes for Supplementary Figure 21, and 22.

Fluorescence Micrograph Acquisition and FITC-PyK Synthesis. Fluorescence micrograph was acquired from a confocal microscope (STELLARIS 5, Leica). The spatially resolved intensity plot was obtained using ImageJ. To synthesize fluorescein labeled pyruvate kinase (FITC-PyK), we use Biotium protocol.[8] We first prepare a solution of 2.5 mg mL^{-1} of PyK in 0.1 M sodium buffer solution (pH 8.5). Then, the dye stock solution was separately prepared by dissolving 10 mM NHS-Fluorescein in anhydrous DMSO. Next, 25 μL of dye stock solution was drop-wise injected into a vial containing 1 mL of PyK stock solution and gently stirred at 600 rpm in the dark (24 °C) for at least 3 h. To remove the unreacted dye, we use a PD-10 column (Cytiva) equilibrated with 0.1 M HEPES buffer. After removal of the unreacted dye by collecting the first band, we dialyzed overnight at room temperature using Slide-A-Lyzer Dialysis Cassette, 10K MWCO (ThermoFisher) for further purification. Concentration of purified FITC-PyK stock solution is calculated by utilizing Bradford assay using Bovine Serum Albumin (BSA) as the standard protein, yielding 1.43 ± 0.11 mg mL^{-1} for the FITC-PyK stock solution. This stock solution was stored at 4 °C until incorporation in the inner aqueous phase of polymersomes. During preparation, 1.43 ± 0.11 mg mL^{-1} FITC-PyK stock solution was diluted in 20.0 mM PEG, 180 mM KCl, 30 mM HEPES (pH 7.4), 6 mM $MgCl_2$ by a volume ratio of 1:5, leading to final concentrations of 0.24 mg mL^{-1} FITC-PyK, 16.7 mM PEG, 150 mM KCl, 25 mM HEPES (pH 7.4), and 5 mM $MgCl_2$.

Determination of FITC-PyK Concentration Using Bradford Assay. We use Bradford assay to calculate the concentration of synthesized FITC-PyK, as previously reported by others and described in the manufacturer's protocol.[9-11] Briefly, BSA (Bovine Serum Albumin, Sigma-Aldrich) was used as the standard, and Bradford reagent (Product No. B6916, Sigma-Aldrich) was used to measure absorbance (595 nm, and 450 nm) for the two protein samples, BSA and FITC-PyK, respectively. First, we prepared a BSA stock solution of 1 mg mL^{-1} in deionized water. Diluted BSA solutions with five different concentrations (0.1, 0.2, 0.4, 0.6, 1 mg mL^{-1}) were prepared from the BSA stock solution and deionized water without BSA was used as the blank. We use 96-well plate and measure absorbance ratio between 595 nm and 450 nm for

linearization[10] using a Hidex Sense Microplate Reader (Hidex, Finland) at 18.8 °C. After dispensing 5 μL of standard BSA and FITC-PyK samples into each microplate well, 250 μL of Bradford reagent is added to each well, mixed for 30 s, followed by incubation at room temperature for 10 min. After obtaining standard curve from the BSA samples (Supplementary Fig. 22), we can determine the FITC-PyK concentration from the measured absorbance ratio.

Note for Supplementary Figure 23. To investigate the enzymatically induced coacervation process in polymersomes, we measure the number of coacervate droplets and their dimensions after PEP infusion as a function of time. Coacervation process is commenced by injection PEP at the periphery of polymersomes whose membrane allow PEP diffusion into the lumen. The formed coacervate droplets gradually merge into a single large droplet approximately after 120 min and increase up to 20.9 ± 1.8 μm in diameter after 3 h.

Note for Supplementary Figure 26. Actin polymerization process is commenced by injection of PEP, K^+, and Mg^{2+} at the periphery of polymersomes whose membrane allow these molecules to diffuse into the lumen. To observe the actin polymerization shown in Supplementary Fig. 25 and 26, we use a confocal microscope (STELLARIS 5 Confocal Microscope, Leica) equipped with a HC PL 10X and 20X Plan apochromatic objective. Confocal micrographs were obtained using 488 nm OPSL CW laser, and 561 nm DPSS laser lines to excite Alexa Fluor 488 Phalloidin and Nile Red respectively, and were analyzed using a LAS X Software (Leica).

Note for Supplementary Figure 27. We prepared two sets of analogous polymersomes with comparable size but with different composition to demonstrate that the membrane permeability can be altered to precisely regulate small substrates without the use of biopores. In the first set of polymersomes, we replaced a portion of Pluronic L121 with Pluronic L61 (PEO_2-PPO_{30}-PEO_2, MW ~ 2000) which exhibit similar *f* value but smaller molecular weight (MW) than Pluronic L121 (MW ~ 4400) by preparing a mixture of 75 mol% Pluronic L121 and 25 mol% Pluronic L61 with a total of 20 wt% polymer dissolved in a mixture of chloroform and cyclohexane (36:64 vol%). This is anticipated to reduce the membrane thickness and thus increase the membrane permeability, as similarly reported by others.[1] For the other set of polymersomes, we used poly(butadiene)-b-poly(ethylene oxide) (PB-PEO) block copolymer as

the membrane constituent, which has been reported to have a higher f value (~ 0.4),[12] and a thicker membrane.[13, 14] Then, we compared the normalized fluorescence intensity change within these sets of polymersomes over time for two fluorescent dye molecules with MW near 500 Da, rhodamine 6G (479 Da) and HPTS (524 Da), respectively.

We observed that the permeability of R6G (479 Da) in Pluronic L121+L61 blended polymersomes is slightly higher (6.93 nm/s) than the analogous Pluronic L121 only polymersomes (6.28 nm/s). Moreover, we found that the PB-PEO polymersomes with higher f value and a thicker membrane exhibit low molecular permeability (0.025 nm/s), which was even lower than the permeability of HPTS in Pluronic L121 polymersomes. Furthermore, similar tendency was observed for HPTS (524 Da) in which we observed increase in HPTS permeability by approximately two-folds from 0.051 to 0.096 nm/s by blending with Pluronic L61 and extremely low permeability for PB-PEO polymersomes (0.016 nm/s).

Supplementary References

[1] do Nascimento, D. F., Arriaga, L. R., Eggersdorfer, M. Ziblat, R. Marques, M. F. V., Reynaud, F., Koehler S. A. & Weitz, D. A. Microfluidic Fabrication of Pluronic Vesicles with Controlled Permeability. *Langmuir* **32,** 5350-5355 (2016)

[2] Allnatt, A. R. Thoery of Phenomenological Coefficients in Solid-State Diffusion. I. General Expressions. *J. Chem. Phys.* **43**, 1855 (1965)

[3] Chabanon, M., Ho, J. C. S., Liedberg, B., Parikh A. N. & Rangamani, P. Pulsatile Lipid Vesicles under Osmotic Stress. *Biophys. J.* **112**, 1682-1691 (2017)

[4] Kedem, O. & Katchalsky, A. A Physical Interpretation of the Phenomenological Coefficients of Membrane Permeability. *J. Gen. Physiol.* **45**, 143-170 (1961)

[5] Lee, H., Choi, C.-H., Abbaspourrad, A., Wesner, C., Caggioni, M., Zhu, T., Nawar, S. & Weitz, D. A. Fluorocarbon Oil Reinforced Triple Emulsion Drops. *Adv. Mater.* **28**, 8425-8430 (2016).

[6] Abbas, M., Lipiński, W. P., Wang, J. & Spruijt, E. Peptide-Based Coacervates As Biomimetic Protocells. *Chem. Soc. Rev.* **50,** 3690-3705 (2021).

[7] Nakashima, N. K., André, A. A. M. & Spruijt, E. Enzymatic Control over Coacervation. *Meth. Enzymol.* **646**, 353-389 (2021).

[8] Protocol: Succinimidyl Ester Labeling of Protein Amines, Biotium, (2020) URL: https://biotium.com/tech-tips/protocol-succinimidyl-ester-labeling-of-protein-amines/

[9] He, F. Bradaford Protein Assay. *Bio-101.* e45. (2011). URL:10.21769/BioProtoc.45

[10] Bradford, M. M. A Rapid and Sensitive Method for the Quantitation of Microgram Quantities of Protein Utilizing the Principle of Protein-Dye Binding. *Anal. Biochem.* **72**, 248-254 (1976).

[11] Zor, T. & Selinger, Z. Linearization of the Bradford Protein Assay Increases Its Sensitivity: Theoretical and Experimental Studies. *Anal. Biochem.* **236**, 302-308 (1996).

[12] Habel, J., Ogbonna, A, Larsen, N., Cherré, S., Kynde, S., Midtgaard, S. R., Kinoshita, K., Krabbe, S., Jensen, G. V., Hansen, J. S., Almdal, K. & Hèlix-Nielsen, C. Selecting analytical tools for characterization of polymersomes in aqueous solution. *RSC Adv.* **5**, 79924-79946 (2015).

[13] Lim, S. K., De Hoog, H.-P., Parikh, A. N., Nallani, M. & Liedberg, B. Hybrid, Nanoscale Phospholipid/Block Copolymer Vesicles. *Polymers* **5**, 1102-1114 (2013).

[14] Seo, H., Nam, C., Kim, E., Son, J. & Lee, H. Aqueous Two-Phase System (ATPS)-Based Polymersomes for Particle Isolation and Separation. *ACS Appl. Mater. Interfaces* **12**, 55467-55475 (2020).
