# Supplementary material for: Spatiotemporal control of signal-driven enzymatic reaction in artificial cell-like polymersomes
Source: Nat Commun. 2022 Sep 2;13:5179. doi: 10.1038/s41467-022-32889-7 (PMC9440086; doi:10.1038/s41467-022-32889-7)
Supplement: Supplementary file 3 — Description of Additional Supplementary Files [file 41467_2022_32889_MOESM3_ESM.pdf]

Supplementary Movie 1: Video of microfluidic synthesis of polymersomes through a glass capillary microfluidic device.
