# Supplementary material for: Spatiotemporal control of signal-driven enzymatic reaction in artificial cell-like polymersomes
Source: Nat Commun. 2022 Sep 2;13:5179. doi: 10.1038/s41467-022-32889-7 (PMC9440086; doi:10.1038/s41467-022-32889-7)
Supplement: Supplementary file 5 — Reporting Summary [file 41467_2022_32889_MOESM5_ESM.pdf]

nature portfolio

Corresponding author(s): Hyomin Lee

Last updated by author(s): Aug 12, 2022

Reporting Summary

Nature Portfolio wishes to improve the reproducibility of the work that we publish. This form provides structure for consistency and transparency in reporting. For further information on Nature Portfolio policies, see our Editorial Policies and the Editorial Policy Checklist.

Statistics

For all statistical analyses, confirm that the following items are present in the figure legend, table legend, main text, or Methods section.

n/a	Confirmed	
☐	☒	The exact sample size (*n*) for each experimental group/condition, given as a discrete number and unit of measurement
☐	☒	A statement on whether measurements were taken from distinct samples or whether the same sample was measured repeatedly
☒	☐	The statistical test(s) used AND whether they are one- or two-sided<br>*Only common tests should be described solely by name; describe more complex techniques in the Methods section.*
☒	☐	A description of all covariates tested
☒	☐	A description of any assumptions or corrections, such as tests of normality and adjustment for multiple comparisons
☐	☒	A full description of the statistical parameters including central tendency (e.g. means) or other basic estimates (e.g. regression coefficient) AND variation (e.g. standard deviation) or associated estimates of uncertainty (e.g. confidence intervals)
☒	☐	For null hypothesis testing, the test statistic (e.g. *F*, *t*, *r*) with confidence intervals, effect sizes, degrees of freedom and *P* value noted<br>*Give P values as exact values whenever suitable.*
☒	☐	For Bayesian analysis, information on the choice of priors and Markov chain Monte Carlo settings
☒	☐	For hierarchical and complex designs, identification of the appropriate level for tests and full reporting of outcomes
☒	☐	Estimates of effect sizes (e.g. Cohen's *d*, Pearson's *r*), indicating how they were calculated

Our web collection on statistics for biologists contains articles on many of the points above.

Software and code

Policy information about availability of computer code

Data collection	Only commercial software was used for data collection: Microsoft Excel 2016, Wallac 1420 Victor2 Microplate Reader Software version 3.00 Revision 5 (PerkinElmer), Hidex Sense Microplate Reader Software 425-301 (Hidex, Finland), confocal microscope systems and LAS X software version 4.1.0 (STELLARIS 5 Confocal Microscope, Leica), NIS-Elements Basic Research 4.60 Software, Photron FASTCAM Viewer 3 Software, TCapture software version 4.3 (Tucsen) were used.
Data analysis	No customized code was used to analyze data. Data were analyzed in Microsoft Excel 2016, Origin Pro 8.0, and ImageJ 2018.

For manuscripts utilizing custom algorithms or software that are central to the research but not yet described in published literature, software must be made available to editors and reviewers. We strongly encourage code deposition in a community repository (e.g. GitHub). See the Nature Portfolio guidelines for submitting code & software for further information.

Data

Policy information about availability of data

All manuscripts must include a data availability statement. This statement should provide the following information, where applicable:

- Accession codes, unique identifiers, or web links for publicly available datasets
- A description of any restrictions on data availability
- For clinical datasets or third party data, please ensure that the statement adheres to our policy

The generated data supporting the findings of this study are provided in the paper, Supplementary Information and uploaded on Figshare (DOI: 10.6084/m9.figshare.20462466). Source Data file are provided with this paper.

Field-specific reporting

Please select the one below that is the best fit for your research. If you are not sure, read the appropriate sections before making your selection.

- [x] Life sciences
- [ ] Behavioural & social sciences
- [ ] Ecological, evolutionary & environmental sciences

For a reference copy of the document with all sections, see nature.com/documents/nr-reporting-summary-flat.pdf

Life sciences study design

All studies must disclose on these points even when the disclosure is negative.

Sample size	The sample size was equal or larger than 5 in all cases. No additional method was used to calculate the sample size. Sample size in the number of plate wells/polymersomes/coacervate droplets for in vitro microscopy imaging was analyzed technically triplicate that can reduce variability and sufficiently confirm the consistency.
Data exclusions	None.
Replication	All experimental data were sufficiently repeated at least three times, and all data points are shown in the manuscript.
Randomization	Randomization was not relevant to this work as our methods did not include medical or clinical trials, and associated experimental analysis.
Blinding	Blinding was not relevant to this work, as our works did not include clinical trials and associated experimental analysis.

Reporting for specific materials, systems and methods

We require information from authors about some types of materials, experimental systems and methods used in many studies. Here, indicate whether each material, system or method listed is relevant to your study. If you are not sure if a list item applies to your research, read the appropriate section before selecting a response.

Materials & experimental systems

n/a	Involved in the study	
[x]	[ ]	Antibodies
[x]	[ ]	Eukaryotic cell lines
[x]	[ ]	Palaeontology and archaeology
[x]	[ ]	Animals and other organisms
[x]	[ ]	Human research participants
[x]	[ ]	Clinical data
[x]	[ ]	Dual use research of concern

Methods

n/a	Involved in the study	
[x]	[ ]	ChIP-seq
[x]	[ ]	Flow cytometry
[x]	[ ]	MRI-based neuroimaging
